# Leveraging mHealth to Mitigate the Impact of COVID-19 in Black American Communities: Qualitative Analysis

**DOI:** 10.2196/47294

**Published:** 2023-12-22

**Authors:** Kelly M Harris, Tilicia Mayo Gamble, Madelyn G Yoo, Lindsay A Spell, Timira N Minor, Holly Jones, Donald Lynch

**Affiliations:** 1 School of Medicine Washington University in St. Louis Saint Louis, MO United States; 2 Department of Health Policy and Community Health Georgia Southern University Statesboro, GA United States; 3 Martha S Pitzer Center for Women, Children, and Youth College of Nursing Ohio State University Columbus, OH United States; 4 Division of Cardiovascular Health and Disease Department of Medicine University of Cincinnati Cincinnati, OH United States

**Keywords:** COVID-19, mobile health, mHealth, information-seeking behavior, Black communities, cardiovascular health, community, qualitative analysis, morbidity, mortality, develop, pilot, evaluate, mHealth intervention, cardiovascular, racism, health equity

## Abstract

**Background:**

COVID-19 remains an ongoing public health crisis. Black Americans remain underrepresented among those vaccinated and overrepresented in both COVID-19 morbidity and mortality. Medical misinformation, specifically related to COVID-19, has exacerbated the impact of the disease in Black American communities. Communication tools and strategies to build relationships and disseminate credible and trustworthy diagnostic and preventative health information are necessary to improve outcomes and equity for historically oppressed populations.

**Objective:**

As the initial phase of a larger mixed methods project to develop, pilot, and evaluate a mobile health (mHealth) intervention among a population at high risk for COVID-19 and cardiovascular comorbidities, this study sought to explore COVID-19 information behavior among Black Americans. Specifically, this study examined (1) preferences for COVID-19 education via mHealth, (2) barriers and facilitators to COVID-19 education and diagnostic testing and routine care for associated cardiovascular and respiratory comorbidities in the local community, and (3) key content for inclusion in a COVID-19 mHealth app.

**Methods:**

This qualitative study used principles of community-based participatory research and information systems research to conduct 7 focus groups across 3 sites. Focus groups were audio recorded and transcribed for thematic analysis using an abductive approach.

**Results:**

The study sample included 54 individuals across sites with a mean age of 50.24 (SD 11.76; range 20-71) years. Participants were primarily female (n=42, 78%) and Black (n=54, 100%) with varied education levels. Over half (n=29, 54%) of the participants were employed full-time, and nearly three-fourths (n=40, 74%) had household incomes <US $65,000. Participants used both Android (n=23, 43%) and iOS devices (n=29, 54%) and were “very comfortable” (n=37, 69%) using their mobile devices. Participants reported using a variety of sources for health information. Content-related preferences reported focus on visual presentation, user-friendly design, and privacy and highlighted the importance of community relevance, access, and community-specific content. Key barriers identified included health literacy–limiting app use, access to technology and information, and lack of trust. Increasing community relevance through community-specific messaging and the inclusion of Black providers were noted as facilitators that may increase credibility and trust. Key content identified included user-specific information such as where to get vaccines and tests, updated local COVID-19 data, travel protocols, information about long COVID-19 (post COVID-19 condition), comorbidities, frequently asked questions, and testimonials or personal stories.

**Conclusions:**

Increasing transparency and building trust are 2 key strategies that may improve the impact of health information messaging in Black communities. Focusing on content over context fails in the provision of critical health information and perpetuates health inequities by reinforcing systemic and structural racism. COVID-19 messaging must consider contextual information, patient needs and preferences, and patient information-seeking and information-search behaviors to establish trust and credibility, positively impact patient health outcomes, and improve health equity.

## Introduction

### Background

Due to the persistent presentation of variants, COVID-19 is an ongoing public health crisis. As the United States navigates COVID-19 variants, individuals who have not received any booster dose are at higher risk of infection, and those who remain unvaccinated are at increased risk for infection, severe illness, and death. Black and Latino Americans are 2 times more likely than White Americans to be hospitalized for COVID-19 and almost twice as likely to pass away, exacerbating racial and health inequities already present in the United States [1]. Over the course of the vaccination rollout, Black Americans have been less likely to receive a vaccine than their White counterparts, despite having significantly higher rates of cardiovascular risk factors and comorbidities associated with worse outcomes in COVID-19 [2]. Unfortunately, this has translated to significantly higher rates of infection among Black Americans and a nearly 2-fold higher risk of dying due to COVID-19 [1].

The parallel pandemic of medical misinformation has compounded the morbidity and mortality of COVID-19 [3,4]. While internet health information seeking is common among adults in the United States, unfortunately, misinformation exacerbates the ongoing challenges of getting medical information into Black communities [5]. Black Americans experience poor communication with their health care providers, medical mistrust, and perceived discrimination when accessing health care in numerous, and sometimes interrelated, ways [6,7]. Hence, it is essential to build trust and acceptance of health recommendations, such as COVID-19 vaccination, among Black communities. Strategies are needed to engage trusted messengers in a meaningful way to lead to sustainable action and partnership [8]. One promising strategy is to partner with faith-based leaders, a highly trusted resource and frequent central gathering place for communities composed of racial and ethnic minority populations [9,10].

Collaborating with faith-based leaders is an approach that has been adopted to leverage mobile health (mHealth) apps to disseminate cardiovascular health information to Black communities [11]. In a recent study, partnering with faith-based leaders led to the advancement of an efficacious mHealth tool to promote cardiovascular health among Black Americans [11]. Thus, partnering with faith-based leaders to develop an mHealth tool to offer COVID-19 and cardiovascular health information could be a channel for addressing 2 major health areas of concern among Black Americans. Moreover, acquiring input from Black American communities for diagnostic, preventative, and intervention measures can shed light on the communities’ multilevel health challenges. The potential use of engaging the faith-based community to facilitate COVID-19 education and diagnostic testing in Black communities remains unknown. We believe that using digital media, such as an mHealth app, to deliver targeted and accurate information at an individual level is essential. Among Black Americans, this study aimed to explore (1) COVID-19 information behavior and preferences for a COVID-19 education via mHealth targeting; (2) barriers and facilitators to COVID-19 education and diagnostic testing and routine care for associated cardiovascular and respiratory comorbidities in the local community; and (3) key content for inclusion to develop an mHealth app to provide COVID-19 education and awareness information and electronic screening tools for COVID-19, hypertension, chronic respiratory disease, and cardiovascular disease (CVD).

### Theoretical Framework: A Nested Model of COVID-19 Information Seeking

**Figure 1:**
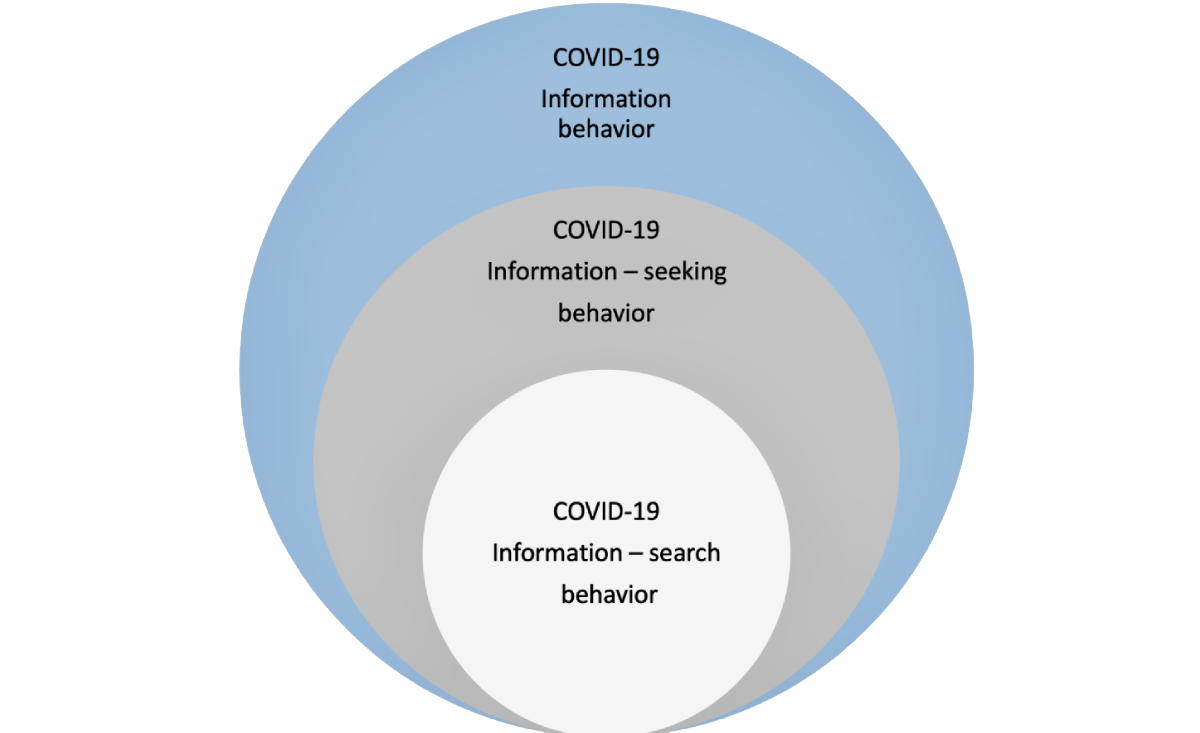
An adapted nested model of COVID-19 information behavior.

The field of communication has a long tradition of studying health information–seeking behavior that focuses on how people seek and manage information about their health [5]. An adapted model of nested information-seeking behavior (Figure 1) influenced this study [12]. While there are existing models describing health information seeking, many of these models are specific to an audience of patients and few examine information seeking in the context of a pandemic [13-15]. To develop an app that would fulfill health information needs related to COVID-19, there was a need to apply a model that would offer a broad understanding of the approaches used to find information on COVID-19 among the general public. According to the adapted model, COVID-19 information behavior is the broad general area of study, COVID-19 information–seeking behavior is a subset of COVID-19 information behavior, and COVID-19 information–search behavior is a subset of COVID-19 information–seeking behavior. Using an inductive-deductive approach, we sought to understand overall COVID-19 information behavior or how individuals sought, received, and processed COVID-19 information. This includes COVID-19 messages received, concerns about the COVID-19 information received, and the importance of finding credible COVID-19 information. Guided by this adapted nested model of information-seeking behavior, this study sought to examine individual COVID-19 information–search behaviors (the interactions between individuals seeking information and the information systems and environments) that are nested within COVID-19 information–seeking behavior (the methods individuals use to find and access information) to better understand the overall COVID-19 information behavior. We also sought to understand barriers and facilitators to COVID-19 information in the context of an individual’s COVID-19 information behavior to inform the development of an mHealth app to increase COVID-19 education and diagnostic testing in Black communities.

## Methods

### Study Context

Three geographical areas were targeted and used for recruitment and data collection due to having disproportionate cardiovascular risk factors impacted by COVID-19: St. Louis, Missouri; Cincinnati, Ohio; and Statesboro, Georgia. The first location, St. Louis, Missouri, is a midsize city (population of approximately 287,000) experiencing stark racial and economic segregation with associated health care access and outcomes disparities [16]. Despite representing only 45% of the population, Black residents in St. Louis visit the emergency department for chronic conditions at 3.5 times that of White residents and experience mortality rates nearly 1.5 times that of White residents [16-18]. COVID-19 death rates for Black residents in St. Louis are nearly twice that for White residents [15,19]. The second location, Cincinnati, Ohio, is another midsize city (population of approximately 310,000) with Black residents having the highest confirmed cases of COVID-19 and 34% higher rates of death despite comprising only 40% of the population identifying as Black [16,20]. The third location, Statesboro, is a rural area in southeast Georgia (population of approximately 34,000) [16]. Statesboro has a large Black community (41%) that experienced increasing rates of COVID-19 throughout the pandemic [16,21].

### Recruitment and Data Collection

The analysis presented here focuses on the initial qualitative phase of a larger-scale mixed methods study to develop, pilot, and evaluate an mHealth intervention among a population at high risk for COVID-19 and associated cardiovascular risk factors. Data collection included 7 focus groups with 54 stakeholders across the 3 targeted locations. To establish a primary stakeholder team, we used new and existing connections with faith-based organizations and purposive sampling to identify and recruit community members for participation in focus groups.

Focus group protocol development was guided by principles of community-based participatory research and Information System Research (ISR). ISR is an iterative process incorporating end-user co-design to build or design products and is effective for mHealth app development [22]. ISR traditionally includes 3 research cycles [22]. First, the relevance cycle is a series of 2 to 4 focus groups designed to develop an understanding of the end-user environment or context. Data collection focuses on identifying what is significant for inclusion, the manner of incorporation, and general user interface preferences. These data then inform app development in the next cycle, prototype design. Following prototyping, evaluation determines which features and components are functional, acceptable, and preferred. Continued iterations refine, evaluate, and finalize the design. This study includes data collected in the first research cycle, the relevance cycle.

### Analytic Approach

This qualitative data corpus included data collected from all 7 focus groups. All focus groups were conducted via Zoom (Zoom Video Communications), audio recorded, and transcribed verbatim. A combined abductive approach, using both deductive and inductive thematic analysis, was used to code the data corpus. Research questions were applied to guide the initial codebook development, followed by inductive analysis of a sample of the transcripts to identify themes that emerged organically for inclusion in the codebook. After comparing and adding emerging themes, the codebook was finalized and used to code all transcripts.

### Ethical Considerations

All study activities and data collection were approved through the institutional review board of each participating institution: Washington University in St. Louis (202011144), the University of Cincinnati (2020-1189), and Georgia Southern University (H21151).

## Results

### Sample

As of July 2021, we recruited and enrolled 54 individuals across all 3 sites for participation in this study. Characteristics of focus group participants are presented in Table 1. Across the 3 sites, the mean age of focus group participants was 50.24 (SD 11.8; range 20-71) years. Participants were primarily female (n=42, 78%) and Black (n=54, 100%). The education level of participants varied with 33% (n=18) having some college degree, 28% (n=15) having an undergraduate degree, and 28% (n=15) having completed postgraduate work. Over half (n=29, 54%) of the participants were employed in full-time positions, and 19% (n=10) were unemployed. In total, 7 (13%) participants were retired. The vast majority of participants had household incomes below US $65,000 (n=40, 74%), with 22% (n=12) having reported a household income of less than US $10,000. Most participants (n=28, 52%) reported being single, and 35% (n=19) were married. Cell phone type varied, with under half (n=23, 43%) reporting the use of an Android device and just over half (n=29, 54%) reporting the use of an iOS device. When asked about comfort connecting to and accessing the internet on their mobile devices, most participants (n=37, 69%) reported that they were “very comfortable” (Multimedia Appendix 1).

**Table 1:** Sample characteristics.

Variable			All sites (N=54)		St. Louis (n=15)		Cincinnati (n=14)		Statesboro (n=25)
**Age (years)**									
	Mean (SD)	50.2 (11.8)		46.1 (14.0)		66.3 (3.3)		38.8 (13.6)	
	Range	20-71		24-64.9		62-71		20-66	
**Sex, n (%)**									
	Female	42 (78)		10 (67)		11 (79)		21 (84)	
	Male	11 (20)		4 (27)		3 (21)		4 (16)	
	I prefer not to say	1 (2)		1 (7)		0 (0)		0 (0)	
**Race, n (%)**									
	Black	54 (100)		15 (100)		14 (100)		25 (100)	
**Education, n (%)**									
	Some high school	0 (0)		0 (0)		0 (0)		0 (0)	
	High school graduate	4 (7)		2 (13)		2 (14)		0 (0)	
	Some college	18 (33)		3 (20)		6 (43)		9 (36)	
	Undergraduate degree	15 (28)		5 (32)		2 (14)		8 (32)	
	Postgraduate work or degree	15 (28)		5 (33)		3 (21)		7 (28)	
**Employment status, n (%)**									
	Unemployed	10 (19)		6 (40)		2 (14)		2 (8)	
	Part-time	6 (11)		0 (0)		2 (14)		4 (16)	
	Full-time	29 (54)		8 (53)		5 (36)		16 (64)	
	Retired	7 (13)		1 (7)		4 (29)		2 (8)	
	Full-time student	0 (0)		0 (0)		0 (0)		0 (0)	
**Income (US $), n (%)**									
	<10,000	12 (22)		6 (40)		4 (29)		2 (8)	
	10,001-25,000	6 (11)		1 (7)		0 (0)		5 (20)	
	25,001-35,000	8 (15)		1 (7)		4 (29)		3 (12)	
	35,001-45,000	9 (17)		3 (20)		0 (0)		6 (24)	
	45,001-55,000	2 (4)		0 (0)		2 (14)		0 (0)	
	55,001-65,000	3 (6)		1 (7)		0 (0)		2 (8)	
	>65,000	12 (22)		3 (20)		3 (21)		6 (24)	
**Marital status, n (%)**									
	Single or not in a relationship	17 (32)		6 (40)		2 (14)		9 (36)	
	Single in a relationship	11 (20)		6 (40)		0 (0)		5 (20)	
	Married	19 (35)		2 (13)		9 (64)		8 (32)	
	Separated divorced	3 (6)		1 (7)		0 (0)		2 (8)	
	Widowed	2 (4)		0 (0)		2 (14)		0 (0)	
**Cell phone type, n (%)**									
	Android	23 (43)		8 (53)		7 (50)		8 (32)	
	iOS	29 (54)		7 (47)		6 (43)		16 (64)	

### COVID-19 Information Behavior

#### Overview

**Figure 2:**
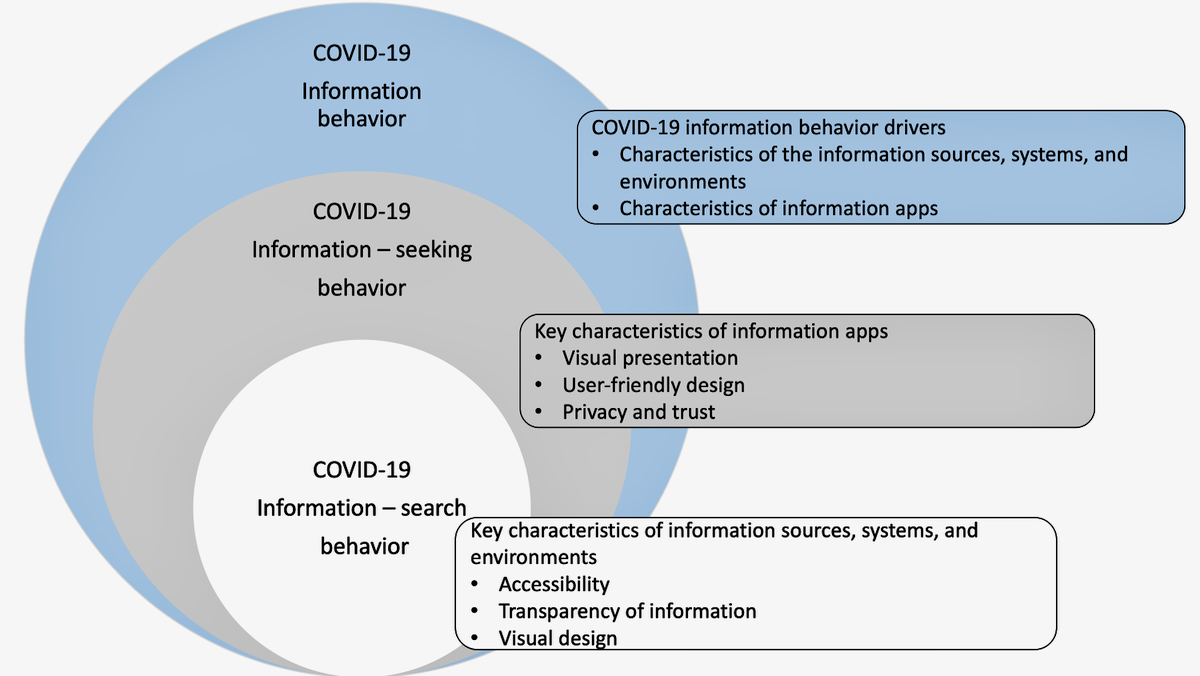
Factors influencing COVID-19 information behavior.

The two primary drivers of COVID-19 information behavior and preferences for a COVID-19 education via mHealth targeting participants described were (1) characteristics and accessibility of information sources, ranging from individual professionals to media outlets and work or government resources, and (2) characteristics of information apps or their individual app preferences (preferences for content and structure). Characteristics of information sources, systems, and environments were central to participant COVID-19 information–search behavior, while characteristics of information apps or individual app preferences informed participant’s COVID-19 information–seeking behaviors (Figure 2).

#### Information Sources, Systems, and Environments

All participants reported using apps on their cell phones (n=54, 100%); however, when asked how they have received information regarding COVID-19, participants reported using a variety of sources for information, and many selected multiple sources, including websites, television, health care providers, peers, and colleagues (Multimedia Appendix 2). When asked to share specific sources of information, responses across all 3 sites ranged from family and friends, health care providers, to media outlets such as Google, CNN, and local news stations. Recognizable websites such as the Centers for Disease Control and Prevention (CDC) website and Johns Hopkins were noted as trusted sources of information, as were work (ie, emails from employers) and government resources such as the local health departments. Individuals actively engaged in the community also listed specific professionals they encountered as indispensable sources of trusted information. For example, 1 participant shared:

I sit on the XXX Advisory—Research Advisory Board. We got access to doctors, so if we got a question, I can call someone from the advisory board. I can call her and say, “Hey, can you ask Dr. XX this question?” She can do it. I can ask Dr. XX. I got access to professionals that can answer my questions.

Cincinnati

#### Information App Preferences

##### Overview

To me, I’ve found that that’s one of the quickest ways for people to get uninterested in what’s going on is when they’re presented with information that a topic they already don’t understand, and then you trying to explain it with words they don’t understand.

St. Louis

Community members across all 3 sites shared key preferences regarding content and structure for formatting of a COVID-19 mHealth app and fundamental things to avoid. Among content-related preferences, participants’ responses fell into three primary thematic categories: (1) visual presentation, (2) user-friendly design, and (3) privacy. Across these 3 themes, participants highlighted the importance of community relevance and access and community-specific content, including images reflective of the community and local and accessible resources as subthemes. One participant specifically suggested:

Showing brown people, Black people, African Americans, and having African American doctors that are giving the information. Because I think that would be maybe a little bit more acceptable to our community.

Statesboro

Another participant suggested using people, content, and modes of delivery (ie, videos and music) that young people or individuals from specific communities identify with or relate to:

...We have to figure out who the young people look up to and maybe have them visually talk about it, do a dance about it, song about it, something...It has to be something they listen to and respect.

Cincinnati

Additionally, a participant from this same site highlighted the tendency of our web-based and mobile resources to prioritize broader events and institutions as opposed to local community-specific content, saying:

There’s no one-stop place that we know about...If you pulled [a google search for local events] up today, you’re only gonna see the Black Family Reunion, the art museum, or something like that. That’s it. Nothing about the community. Nothing.

Cincinnati

##### Visual Presentation

Participants across all 3 sites highlighted the importance of presenting visually appealing content in various modes and locations. Participants noted the importance of imagery as opposed to words to increase accessibility and attract more attention. Participants in 3 of the 7 focus groups reported that using an “eye-catching” platform and images may help accessibility for those with literacy or visual impairments.

Provide pictures with words, as well, just in case—like, within the app itself, provide pictures with some of those words, where somebody can easily look at that picture and maybe know what you’re asking or some of the information that’s being said. Create an audio version of the information, so they can listen if they can’t read.

Statesboro

Participants also found the visual presentation and coordination with the local community context essential for branding and advertising. Participants highlighted the need to share information about the app widely for community members to see it multiple times and in various places. One participant noted:

A lotta times we don’t pay attention to things we see one time or two times. We gotta see it seven times. So, it’s gonna be on the media. It’s gonna have to be on fliers at our churches and our neighborhoods, pharmacies, information about the app. And someone is going to say, “Hey, I’ve seen this before.”

St. Louis

Multiple participants reported the need for “eye-catching” or “attention-grabbing” platforms and materials with limited text and strategic use of visuals as well as video and audio content. Overall, 3 of the 7 focus groups suggested the use of colors and cartoon figures appropriate for adults. For example, one participant noted:

Have it really colorful and pictures of—it don’t really have to be no pictures of real people. Just really bright beautiful colors. [Cincinnati]

Another participant at the same site agreed and suggested this as a way to appeal to both younger and older audiences, noting:

You know some of these commercials like on the Kroger’s commercial, they’re using cartoon characters, and in this other program we’re in, we use the cartoon characters. Something like that, like. said, colorful. You know what I mean...I think that would appeal to both the young and the old ‘cause it’s working on this other program we’re doin.

Cincinnati

##### User-Friendly Design

Participants across all 3 sites in 6 of the 7 focus groups expressed the need for efficient delivery of information and an intuitive, free platform easily accessible by people of varying education levels and age groups. Participants highlighted the importance of avoiding login delays, excess information, and the use of jargon or “wordiness.” One participant noted:

I think it should be user friendly. You know, there’s something that, um, you know, you don’t have to go through a whole bunch of screens and-and options in order to get information.

St. Louis

Participants reported that features such as immediacy, familiarity, and ease of access and use would make the app more desirable. Avoiding requiring users to log in multiple times or to navigate complex menus or screens was also noted as important.

So, something that would be familiar to them, that they’ll know, “This is the right thing that I should be clicking on,” would be very helpful.

Statesboro

Another participant reported that a tab structure would facilitate navigation:

I like that suggestion of actually having individual tabs for different—what we call comorbid medical conditions...it doesn’t force people to go through unnecessary or unneeded content if it’s not necessarily relevant to them.

Cincinnati

Quick access to immediate and relevant information was a common theme across all 3 sites:

Immediacy is what people look for, you know? You have to read a few sentences and get an idea of what’s on the full page, you know? Like speed-reading.

St. Louis

Participants identified specific features that may make the app design user-friendly, including a zoom feature, so that participants can zoom in and out on specific content, push notifications, widgets, QR codes, and single button access to a chat or call feature. Participants also suggested access to up-to-date data on COVID-19 rates and transmission in an easily accessible format.

##### Privacy

In addition to poor user-friendliness, privacy and transparency were highlighted as important areas of concern by participants across all 3 sites. Specifically, participants suggested avoiding third-party apps, requests for personal information, frequent log-in requirements, and asking users several questions. Transparency related to privacy (specifically location tracking and inputting personal information) and the inclusion of app developers and sponsors came up in several areas as highly important to users. Proceeding from the inclusion of sponsors, one participant said:

Absolutely not. I wouldn’t believe a thing that was on there. Even if it’s true I would question it.

Cincinnati

The tracking features used on many apps and websites reoccurred as a frequent concern, as did trust. Participants in 5 of 7 focus groups reported concern with assurance, including ensuring the information presented is accurate and reputable including sources of information trusted by the community, and being transparent by sharing the sources of information. One participant stated:

It’s the trust thing, for me. And I’m so big on, like, “Okay, who are you? What information do you have? Where are you getting it from?”

Statesboro

Some participants connected trust back to the content themes of community relevancy and community specificity. One participant noted:

Now, if it’s a Black, African American descent health care provider, and they really know their facts, I mean, the black-and-white facts, and they’re trusted along the lines and I see them, I would trust them.

Statesboro

Another participant in this same group suggested sharing information about both app sponsors and information content sources clearly and upfront.

Think when you first log in, maybe the app should say, “This app is powered by xyz,” or, “Information is updated by your local county,” or CDC, or, you know, wherever that trusted information is gonna come from, we need to see that updated.

Statesboro

### COVID-19 and CVD Education and Testing Barriers and Facilitators

The second objective examined barriers and facilitators to COVID-19 education and diagnostic testing and routine care for associated cardiovascular and respiratory comorbidities in the local community. Community relevance also appeared as a primary theme in the area of key facilitators. These included the use of information from Black American sources (doctors and health care providers) to increase credibility and trust among their communities and allow members of the community to share their testimony or experience as noted in the quote above.

Several site-specific barriers emerged including challenges with literacy (educational, health, and technological) limiting app use, lack of access to technology and information, and lack of trust. The St. Louis site’s primary concerns were literacy and trust, and the Statesboro site had concerns about all 3 barriers. When discussing access to technology and information, one participant noted:

In rural communities, it’s hard to get information out...especially seniors, don’t have access to the Internet. Some of ‘em don’t have access to newspapers anymore because they’re very rare, and it’s just really hard for rural people to get information that they need...Even in rural areas, they may have access, but it may not pick up where they live...stations or the radio stations...So, they’re suffering because they’re just not getting this information. [Statesboro]

Concerns around trust as a barrier to COVID-19 and CVD education and testing fell into three primary categories: (1) skepticism due to social influences of historical injustice, (2) modern politics and misinformation or political influence clouding the validity of information, and (3) apprehension based on input from family members. In 3 of 7 focus groups, several participants pointed to sentiments of skepticism due to social influences of historical injustice, specifically, referencing the Tuskegee experiment and other historical injustices using Black individuals as “guinea pigs.” One participant connected this to the distrust of vaccines: “...our culture is not willing, not everybody, is willing to go and get a vaccine.” Another participant connected to the overall mistrust of the health care system, stating “...given the history of African Americans and healthcare and how everyone has been doing trials on African Americans, that is where the distrust lies” (Statesboro). A separate participant in this same group connected this distrust to messaging: “I feel like the government or the CDC, maybe they’ve been doing the best they can, but I feel like they have done a horrible job at dispelling a lot of the myths for the African American culture” (Statesboro).

Participants in 2 of 7 focus groups (both at the same site) highlighted that modern politics, specifically, misinformation and political influence, clouds the validity of information. These comments focused on concerns about trusting the legitimacy of information posted on the CDC’s website. For example, one participant noted that while they found themselves on the CDC’s website:

I want to trust them, but at the same time it is some reservation with it. Because I don’t know if you are telling me everything that I should know.

Statesboro

Another participant noted:

Just like the pressure the President put on the CDC. He would not allow certain information to go out. So once, from the top down, you know, misinformation started coming out, then they kind of opened the floodgates from hell to misinformation.

Statesboro

Participants in 2 different groups (at the same site) reflected on hearing information suggesting that Black individuals could not contract COVID-19 and the negative impact this had on communities: “Well, one of the things that’s crazy, in the very early stages of COVID, on some Black radio stations, they were even putting out the information, ‘well, Black people can’t get COVID’” (Statesboro). The third and final barrier noted was apprehension based on input from family members. Those with family members who use different media sources reported receiving conflicting information from family, making them more or less apprehensive regarding COVID-19 testing and vaccines.

### Key Content for Inclusion

The third objective examined key content for inclusion to develop an mHealth app to provide COVID-19 education and awareness information and electronic screening tools for COVID-19, hypertension, chronic respiratory disease, and CVD. Participants in 5 of 7 focus groups (across all 3 sites) requested user-specific information: where to get vaccines and tests, updated COVID-19 case counts in their area, and travel protocols. They also highlighted the importance of access to these data without requiring the disclosure of personal information (ie, location). COVID-19 education about home and day-to-day management of COVID-19 symptoms and what to do when experiencing specific symptoms (ie, quarantine protocols and when to see a physician) were included as key content. While location tracking was cited as a potential barrier, some participants requested the option to see resources located near or closest to them. For example, participants across all 3 sites requested locations of COVID-19 testing and vaccination sites and optional documentation storage for results and vaccination cards. Multiple participants reported the importance of vaccination timing and suggested a calendar or clock for vaccination timing showing when they are due for their next COVID-19 vaccination. Up-to-date data on COVID-19 were a frequent request across all sites, including notifications for updates to CDC guidance and contact numbers for COVID-19 resources.

While cautious about overloading individuals with information, participants suggested information about long COVID-19 (post COVID-19 condition), comorbidities, frequently asked questions, and testimonials or personal stories. Participants suggested this information as a tool to learn about risk and a source of social or emotional support. Participants highlighted the need to be able to access specific relevant information about COVID-19 and individual risk, for example:

If there’s a frequently asked questions tab, and it says on there, “the effects of obesity on COVID-19; the effects of diabetes and COVID-19. Then they can press on either one that they want. They don’t have to see it all.”

Cincinnati

Additionally, participants across sites highlighted the need for testimonials or personal stories from those who have experienced COVID-19, support groups, or community forums to provide social and emotional support. One participant noted that the app should “provide a lot of information while they’re going through that process, so they don’t feel lost or alone” (Statesboro). Another participant at a different site suggested the app includes “a way people could talk amongst themselves just to get a better understanding, or just kinda voice their thoughts and what they think about the situation” (St. Louis).

## Discussion

### Principal Findings

Prior studies have focused on identifying information-seeking behaviors based on perception of risk but without a focus on at-risk groups or tailored information [15,23,24]. This study builds upon that literature to inform the development of targeted or tailored information. Our results suggest that overall COVID-19 information behavior was driven by characteristics of the sources of information and individual app preferences. Information app preferences informed individual COVID-19 information–seeking behavior or the methods individuals use to find and access information. Specifically, individuals sought accessible and trusted sources of information and apps with visually appealing content, user-friendly design, and those allowing for privacy (not tracking location or asking for personal information, etc). Individual COVID-19 information–search behaviors, or the interactions between individuals seeking information and the information systems and environments, were guided primarily by the characteristics of those information systems and environments, specifically accessibility, transparency, and visual design.

Participants sampled in this study were all Black and aware of racial disparities in health care and outcomes, both in the pre–COVID-19 and COVID-19 context, and structural and systemic factors undergirding these inequities. Responses suggested that this awareness impacted participants’ COVID-19 information–seeking and COVID-19 information–search behavior. The sources sought out and viewed as trusted were heavily influenced by familiarity and community relevance. As the COVID-19 pandemic is ongoing with persistent and continually developing variants, these are factors required for consideration as we identify and expand COVID-19 education, testing, and vaccination strategies. Improving communication in Black communities that have historically been underserved by the health care system requires an understanding of the information-seeking and information-search behavior of Black communities.

Several barriers and facilitators to COVID-19 education and testing emerged and informed participant COVID-19 information behavior. While cell phones were a primary and comfortable source of information, participants reported accessing various sources when seeking COVID-19 information. Familiarity, trust, and the organization providing the information (ie, workplace and government) guided the participants in the selection of sources. Equally important were the actual characteristics of individuals sharing COVID-19 information and relevance to the community targeted with this information. Participants’ information-seeking behavior was influenced by how well they understood the material and whether they could relate to the presented material. Using images that reflect the community and ensuring that information is specifically relevant to the local community are critical design elements for understanding what is meaningful to specific communities and whom they trust.

Visual presentation and user-friendliness were the primary design features guiding participant information-search behavior. Visually appealing content with imagery reflective of the local community is preferred, and ease of use and efficiency, or quick access to relevant information, were also decisive influences on participant search behavior. Privacy and trust were frequent notable concerns. Privacy around app use, particularly third-party apps, requests for personal information, and location tracking were concerns in COVID-19 information. Participants were concerned not only about the trustworthiness of the information presented but also about the credibility of the presenter. The lack of transparency and the inclusion of sponsors were additional factors that impacted the trustworthiness of COVID-19 information received. These findings are consistent with prior literature suggesting that building trust and attending to the needs of specific groups are important strategies to create sustainable partnerships and improve the impact of health information messaging in Black communities [8,13].

Participants were keenly aware of historical injustices, particularly the history of medical racism, and considered this as a factor in both their health-seeking behavior and concerns around the information they received [25-27]. This paired with sentiments of political distrust, and awareness of the increasing prevalence of misinformation heightened these responses. When crafting COVID-19 messaging and health recommendations, we must consider this history and context as well as the prevalence of misinformation as these factors influence health information–seeking behavior and lead to poor communication, particularly in Black communities [6]. Community members may be skeptical of messaging that fails to address the legacy of systemic racism on the health of Black communities and exacerbate the already present health inequities.

Recommendations for key content to include in an app, our third objective, was user- but not site-specific. Participants noted specific information they would find useful but also frequently cautioned against barriers, such as location tracking, that they felt were more important than the content. While individuals noted wanting information specific to their locations, the types of information participants requested were consistent across sites. While this study sought to examine the preferences for the presentation and content of COVID-19 education materials and barriers and facilitators to COVID-19 education and testing, these findings also demonstrate how these factors influence or are influenced by COVID-19 information–seeking behavior.

### Limitations

We recognize that national and local systems control response to disease outbreaks such as COVID-19 and the dissemination of information regarding the spread of diseases. We also recognize that local practices impact health education and other downstream factors resulting from the spread of disease generally and the COVID-19 pandemic specifically. This is a qualitative analysis examining COVID-19 information behavior across 3 sites. While it is not the intent of qualitative work, we realize that the generalizability of our findings is limited. Despite this, this study helps us to understand the values, norms, and standards impacting COVID-19 information behavior among Black Americans. These findings provide novel information informing the development of an app providing relevant COVID-19 education and diagnostic information to underrepresented communities to improve outcomes.

### Conclusions

Not only did this study explore the preferences for barriers and facilitators to COVID-19 education among Black Americans it also identified several factors influencing the COVID-19 information behaviors of the participants. Our findings suggest that focusing on content over context fails the individuals seeking health information, but it also reinforces the systemic and structural racism that perpetuates health inequities and leads to poorer outcomes in Black communities. These implications are relevant both for health education providers and clinicians. Understanding patient needs and preferences for health information as well as their information-seeking and information-search behavior is critical for establishing trust and credibility, providing quality and impactful care, and improving health equity.

## Appendix

Multimedia Appendix 1 Participant comfort with mobile internet use.

Multimedia Appendix 2 Sources of COVID-19 information.

## Abbreviations

CDC: Centers for Disease Control and Prevention
CVD: cardiovascular disease
ISR: Information System Research
mHealth: mobile health
