# Supplementary figures and images for: Leveraging mHealth to Mitigate the Impact of COVID-19 in Black American Communities: Qualitative Analysis
Source: JMIR Hum Factors. 2023 Dec 22;10:e47294. doi: 10.2196/47294 (PMC10770780; doi:10.2196/47294)

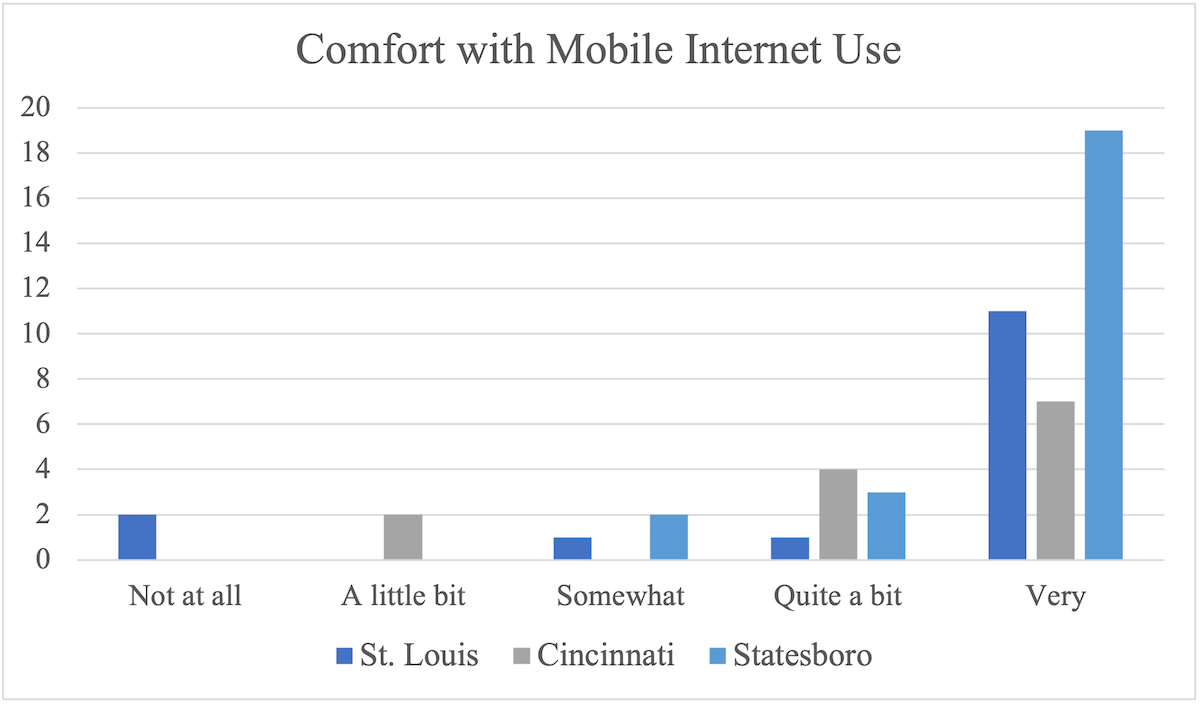

Supplement: Multimedia Appendix 1 [file humanfactors_v10i1e47294_app1.png]

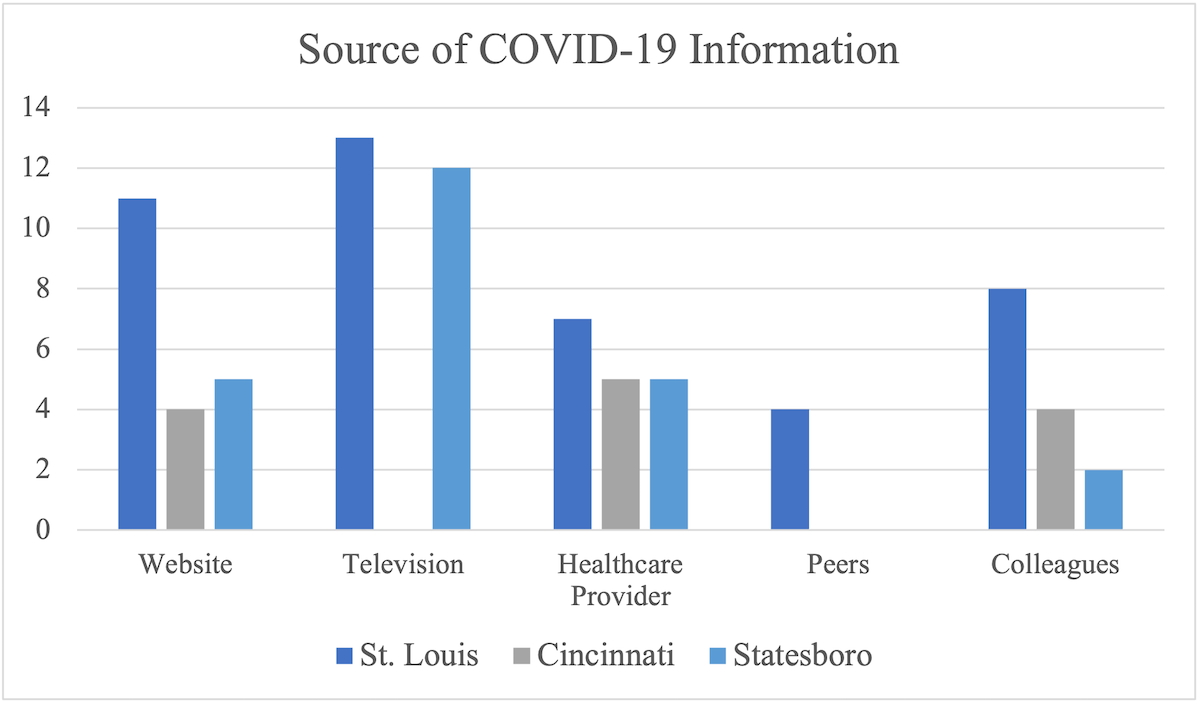

Supplement: Multimedia Appendix 2 [file humanfactors_v10i1e47294_app2.png]
